# Integrative roles of human amygdala subdivisions: Insight from direct intracerebral stimulations via stereotactic EEG

**DOI:** 10.1002/hbm.26300

**Published:** 2023-04-19

**Authors:** Huaqiang Zhang, Di Wang, Penghu Wei, Xiaotong Fan, Yanfeng Yang, Yang An, Yang Dai, Tao Feng, Yongzhi Shan, Liankun Ren, Guoguang Zhao

**Affiliations:** ^1^ Department of Neurosurgery Xuanwu Hospital, Capital Medical University Beijing China; ^2^ School of Engineering Medicine Beihang University Beijing China; ^3^ Beijing Institute of Brain Disorders Beijing China; ^4^ National Medical Center for Neurological Diseases Beijing China; ^5^ Chinese Institute for Brain Research Beijing China; ^6^ China International Neuroscience Institute Beijing China; ^7^ Clinical Research Center of Epilepsy Xuanwu Hospital, Capital Medical University Beijing China; ^8^ Beijing Municipal Geriatric Medical Research Center Beijing China

**Keywords:** electrical stimulation, function, human amygdala, SEEG, subnuclei

## Abstract

Substantial studies of human amygdala function have revealed its importance in processing emotional experience, autonomic regulation, and sensory information; however, the neural substrates and circuitry subserving functions have not been directly mapped at the level of the subnuclei in humans. We provide a useful overview of amygdala functional characterization by using direct electrical stimulation to various amygdala regions in 48 patients with drug‐resistant epilepsy undergoing stereoelectroencephalography recordings. This stimulation extends beyond the anticipated emotional, neurovegetative, olfactory, and somatosensory responses to include visual, auditory, and vestibular sensations, which may be explained by the functional connectivity with cortical and subcortical regions due to evoked amygdala‐cortical potentials. Among the physiological symptom categories for each subnucleus, the most frequently evoked neurovegetative symptoms were distributed in almost every subnucleus. Laterobasal subnuclei are mainly associated with emotional responses, somatosensory responses, and vestibular sensations. Superficial subnuclei are mainly associated with emotional responses and olfactory and visual hallucinations. Our findings contribute to a better understanding of the functional architecture of the human amygdala at the subnuclei level and as a mechanistic basis for the clinical practice of amygdala stimulation in treating patients with neuropsychiatric disorders.

## 1 INTRODUCTION

The amygdala is an almond‐shaped structure found in the cerebral hemisphere of all vertebrates. It comprises a dozen nuclei and is implicated in several specific functions, including stimuli perception, emotion, cognition, and environment‐adapted behavior (Urban & Rosenkranz, 2020). The amygdala has been implicated in several human and animal diseases, such as psychological depression (Linhartová et al., 2019), schizophrenia (Zheng et al., 2019), dementia (Kark & Kensinger, 2019), chronic pain (Thompson & Neugebauer, 2017), and substance abuse (Munshi et al., 2021). Therefore, it is crucial to obtain information on the physiology and function of the amygdala, which is challenging in humans because its role in healthy individuals and the development of specific diseases is complex and critical.

Animal studies have revealed that the mammalian amygdala contains a constellation of subnuclei (Duvarci & Pare, 2014; Sah et al., 2003), which were composed of at least three subdivisions, including the basolateral complex of the amygdala (BLA), medial amygdala, and central amygdala (CeA) (Ehrlich et al., 2009; McDonald, 2003), with different functions and connections. Inter‐nuclei integration and connectivity between subnuclei and the cortical cortex are usually flexible and critical for behavior guidance (Gothard, 2020). The BLA receives sensory information from cortical and thalamic projections, whereas the CeA is the major output of the amygdala (Duvarci & Pare, 2014). Thus, subnuclei‐level research is essential to profile amygdala function.

The human amygdala consists of six subregions based on its cytoarchitecture: the laterobasal group (LB), the superficial group (SF), the centromedial group (CM), the intermediate fiber bundles (IF), the ventromedial part (VTM), and the medial fiber bundles (MF) (Amunts et al., 2020). Several attempts have been made to define the functions of the amygdala subnuclei in human mood, autonomic nervous regulation, and internal states (Barman & Yates, 2017). The BLA and CM are crucial for emotional and social experiences (Izquierdo et al., 2016; Rosenberger et al., 2019), and amygdala subnucleus dysfunction has been linked to several psychiatric disorders (Lowe et al., 2015; Ressler et al., 2011). Furthermore, studies have revealed that BLA structural alternation is associated with pain sensitivity (Zhang et al., 2021), and subnuclei volume alteration has been found in patients with psychiatric disorder (Armio et al., 2020; Zhang et al., 2020). Nevertheless, clinical experience with BLA deep brain stimulation (DBS) results in both positive and negative responses (Avecillas‐Chasin et al., 2020; Langevin, Chen, et al., 2016; Langevin, Koek, et al., 2016). One major issue with varying outcomes may be caused primarily by the complex role of the human amygdala subnuclei. Thus, defining the functions of amygdala subnuclei is fundamental to the amygdala DBS clinical practice.

The explicit role of the human amygdala subnuclei remains a subject of debate, as different data indicate different roles at the subnuclei level (Seguin et al., 2021; Yoder et al., 2015). Direct electrical stimulation is a perturbation technique that allows for more unambiguous brain function delineation than fMRI, diffusion tractography, and pharmacological investigation. Direct stimulation produces simple effects in unimodal brain networks while eliciting heterogeneous and complex responses in heteromodal and transmodal networks. This study aimed to provide a comprehensive understanding of the roles of amygdala subnuclei in the human brain by direct electrical stimulation of the amygdala subnuclei.

## 2 MATERIALS AND METHODS

### 2.1 Patients

We performed a descriptive observational study that, retrospectively, included patients with focal drug‐resistant epilepsy who underwent stereoelectroencephalography (SEEG) exploration in the Department of Neurosurgery, Xuanwu Hospital, Beijing, between 2016 and 2021 to localize the epileptogenic zone before surgery. The SEEG targets were chosen after a multidisciplinary presurgical evaluation. The study included patients with at least one intracerebral electrode implanted in the amygdala, whereas those with amygdala lesions were excluded. Furthermore, because the study focused on the clinical observations elicited by electrical stimulation, any diagnosis of psychiatric disorder or learning disability was excluded because these conditions could influence the evoked subjective perpetual and behavioral phenomena.

Electrical stimulation is routinely used to localize the epileptogenic zone and generate functional profiles in explored regions. All patients were fully informed of the purpose and risks of the SEEG procedure, and informed consent was obtained in accordance with the Declaration of Helsinki. The Institutional Review Board Committee of Xuanwu Hospital, Capital Medical University, China, approved this study.

### 2.2 Surgical procedure and SEEG monitoring

Three‐dimensional T1‐weighted contrast MRI was performed preoperatively to avoid major vessel injury during the design of the SEEG implantation scheme. An epileptologist and a neurosurgeon carefully reviewed the implantation plan. During general anesthesia, multi‐lead SEEG electrodes (Alcis, Besancon, France) with contacts ranging from 5 and 18, 0.8 mm in diameter, 2 mm in length, and 1.5 mm apart, were implanted under the guidance of a stereotactic robot, ROSA (Medtech, Montpellier, France). Following surgery, the patient underwent computed tomography to verify the location of each electrode and to detect any intracranial hemorrhages or other complications that occurred.

All the patients were monitored using video cameras over the long term to capture habitual clinical seizures. In addition, the intracranial EEG was recorded at 2048 Hz using a Nicolet data acquisition system (Natus, Orlando, USA). All recordings were obtained and referenced to a common contact placed subcutaneously.

### 2.3 Reconstruction of SEEG electrodes and localization of contacts

The SEEG electrodes of each patient were then reconstructed within the Montreal Neurological Institute space using Lead‐DBS (www.lead-dbs.org) following a previously described protocol to reveal the electrode location of all patients. SPM12 (http://www.fil.ion.ucl.ac.uk/spm/software/spm12) was used linearly to coregister postoperative CT images with preoperative MRI. Coregistration was manually controlled for each patient and refined if required. The images were then normalized into the ICBM 2009b NLIN asymmetric space using the SyN approach implemented in advanced normalization tools (http://stnava.github.io/ANTs) based on preoperative MRI. The locations of the contacts in the normalized images were checked using raw images. During the procedure, the images were examined slice‐by‐slice from the tip to the end of the electrode in the standard and individual spaces.

### 2.4 Intracranial electrical stimulation protocol

During the SEEG studies, current‐related intracranial stimulation was performed using a Nicolet Cortical Stimulator (Natus, Orlando, USA) as a routine procedure for diagnostic purposes and topographical delimitation of eloquent brain areas that should be considered in subsequent surgical procedures. High‐frequency stimulation (frequency at 50 Hz, pulse duration of 300 μs, train duration of 5 s) was used in the same manner as previously reported (Caruana et al., 2018; Lanteaume et al., 2007; Qi et al., 2022; Winawer & Parvizi, 2016), with a bipolar mode of stimulation to adjacent contacts. Stimulus intensities ranged from 0.5 to 8 mA. Patients were asked to recline, remain restful, calm, and awake before functional mapping. The patients were blinded to the time points at which the stimulation was delivered. An experienced neurologist and neurosurgeon asked the patients to describe any sensations and feelings in their own words, which were then assessed and categorized (Avecillas‐Chasin et al., 2020; So & Alwaki, 2018). Subjective reports and clinical observations elicited by each stimulation were carefully recorded.

All the stimulations evoking a symptom were considered effective, except for those inducing symptoms throughout the propagation of the stimulus to extra‐cerebral areas (e.g., meningeal pain or scalp paresthesia), which were considered non‐effective. The effective electrical stimulations were then classified according to the presence or absence of electrical modification on the EEG, defined as after discharge (AD). The responses to effective stimulations were classified as physiological if never experienced by the patient before and without electrical modification (Supplementary Table 2). Finally, subject reports and clinical observations were classified into the following semiological categories: (i) emotional responses (including positive or negative emotions); (ii) neurovegetative symptoms (including all types of autonomic symptoms); (iii) visual hallucinations; (iv) olfactory hallucinations; (v) somatosensory responses (including numbness, pain, or itching); (vi) auditory symptoms; (vii) vestibular sensations (including dizziness or vertigo); (viii) loss of contact; (ix) automatisms (including oroalimentary or gestural automatisms); (x) motor symptom (including clonic or tonic movements); (xi) racing thoughts; (xii) multimodal response (including complex symptom belonging to more than one of the above categories); and (xiii) unclassified response (when the effect was not possible to include in any of the above categories) (Balestrini et al., 2015; Mariani et al., 2021; Qi et al., 2022; So & Alwaki, 2018).

### 2.5 Statistical analysis

Descriptive statistics of the variable data were analyzed. Quantitative variables are expressed as mean and standard deviation. The frequency distributions describe the evoked responses of each subnucleus. The chi‐square goodness‐of‐fit test was performed for the related samples to evaluate the distribution of evoked responses between the hemispheres. Statistical significance was set at *p* < .05. Statistical analysis was performed using SPSS software version 28 (IBM SPSS, USA).

## 3 RESULTS

### 3.1 Patient demographic and clinical data

Forty‐eight right‐handed patients (22 women and 26 men) were enrolled in the study. The mean age of the patients was 28.1 years. The epileptogenic zone defined by SEEG exploration included the amygdala in 33 (69%, 33/48) patients. Sixteen patients had electrodes implanted in the right amygdala, twenty‐four with electrodes implanted in the left amygdala, and eight with electrodes implanted bilaterally in the amygdala. Table 1 summarizes the demographic and clinical characteristics of the patients in the study. Eighteen (38%, 18/48) patients had amygdala electrodes implanted outside the epileptogenic zone (Supplementary Table 1 shows details of each patient).

**TABLE 1:** Patient demographics and clinical characteristics

Variable		Total numbers
Gender	Female	22
	Male	26
Age (years), mean (standard deviation)		28.1 (9.8)
Onset (years), mean (standard deviation)		13.4 (9.3)
Duration (years), mean (standard deviation)		14.8 (9.0)
Side of EZ	Right	18
	Left	29
	Bilateral	1
Side of SEEG	Right	16
	Left	24
	Bilateral	8
Contacts in amygdala	Right	58
	Left	94
Trials	Right	91
	Left	159

### 3.2 Contact distribution within the amygdala

**FIGURE 1:**
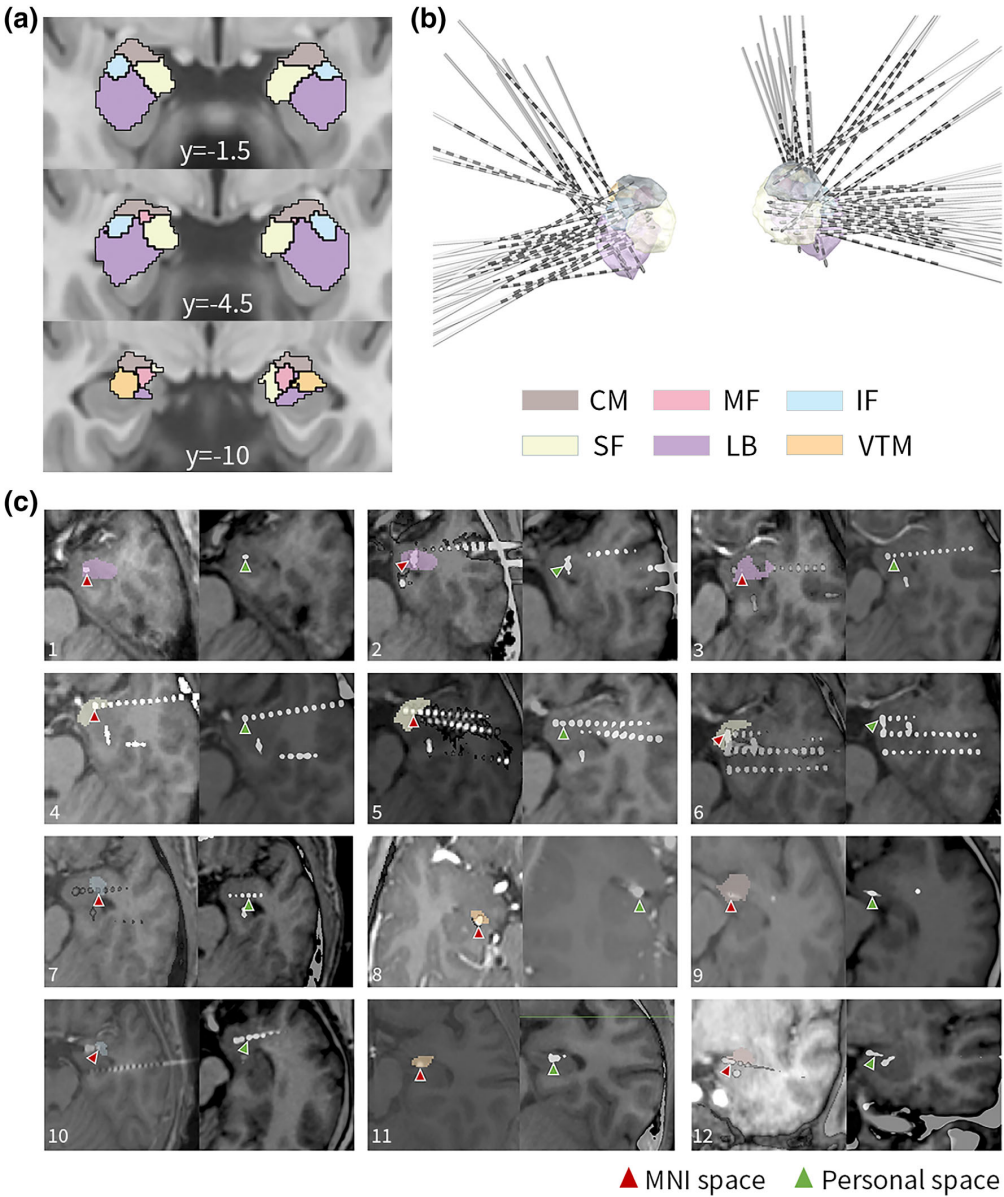
Parcellation of the amygdala overlaid in slices with MNI space and reconstruction of stereoelectroencephalography (SEEG) electrodes in all patients. (a) The amygdala was divided into the centromedial group (CM), medial fiber (MF), intermediate fiber (IF), superficial group (SF), laterobasal group (LB), and ventromedial part (VTM) according to the cytoarchitecture (Julich‐Brain, *Science*, 2020). (b) The trajectories of SEEG electrodes into the amygdala overlaid with all patients in MNI space. (c) Location of some sample contacts of the amygdala subnuclei in the MNI space (red arrow) and the personal space (green arrow). The first row (1–3) shows the contacts within the LB. The second row (4–6) shows the contacts within the SF. The last two rows show the contacts within IF (left column, 7 and 10), VTM (middle column, 8 and 11), and CM (right column, 9 and 12).

There are 81 SEEG electrodes with 152 contacts implanted in the amygdala. Figure 1 depicts the reconstruction of the amygdala electrodes and the anatomical locations of the amygdala subnuclei groups. The amygdala contacts were asymmetrically distributed across the right and left cerebral hemispheres (58 vs. 94, *χ* ^2^, *p* = .004) (Table 1). These amygdala electrodes were revealed to have 90 contacts (59%, 90/152) in the LB, 32 contacts (21%, 32/152) in the SF, 10 contacts (7%, 10/152) in the CM, 14 contacts (9%, 14/152) in the IF, 5 contacts (3%, 5/152) in the VTM, and 1 contact (1%, 1/152) in the MF. The trajectories of some electrodes reaching rarely implanted targets, including the SF, IF, VTM, and CM, are shown in Figure 1c.

### 3.3 Types of evoked responses

Overall, 250 responses elicited by electrical stimulations were observed in the analysis. Of these, 107 (43%, 107/250) elicited physiological symptoms without electrical after‐discharge (AD), 72 (29%, 72/250) elicited symptoms related to habitual ictal symptomatology without pathological electrical activity, and 74 (30%, 74/250) were associated with AD. Multiple responses were evoked according to the clinical response classification. We obtained 46 emotional responses (18%, 46/250), 30 visual hallucinations (12%, 30/250), 4 auditory symptoms (2%, 4/250), 18 olfactory hallucinations (7%, 18/250), 38 somatosensory responses (15%, 38/250), and 125 neurovegetative symptoms (50%, 125/250). Others are 15 vestibular sensations (6%, 15/250), 5 unclassified responses such as vague symptoms (2%, 5/250), 10 episodes of loss of contact (4%, 10/250), 6 motor symptoms (2%, 6/250), and 4 automatisms (2%, 4/250).

### 3.4 Physiological symptoms

Physiological symptoms were observed in 24 patients. Amygdala contacts that elicited physiological symptoms were asymmetrically distributed across the right and left cerebral hemispheres (12 vs. 47, *χ* ^2^, *p* < .001). The location of these contact electrodes revealed 28 contacts (47%, 28/59) in the LB, 15 (25%, 15/59) in the SF, 6 (10%, 6/59) in the CM, six (10%, 6/59) in the IF, and 4 (7%, 4/59) in the VTM.

**FIGURE 2:**
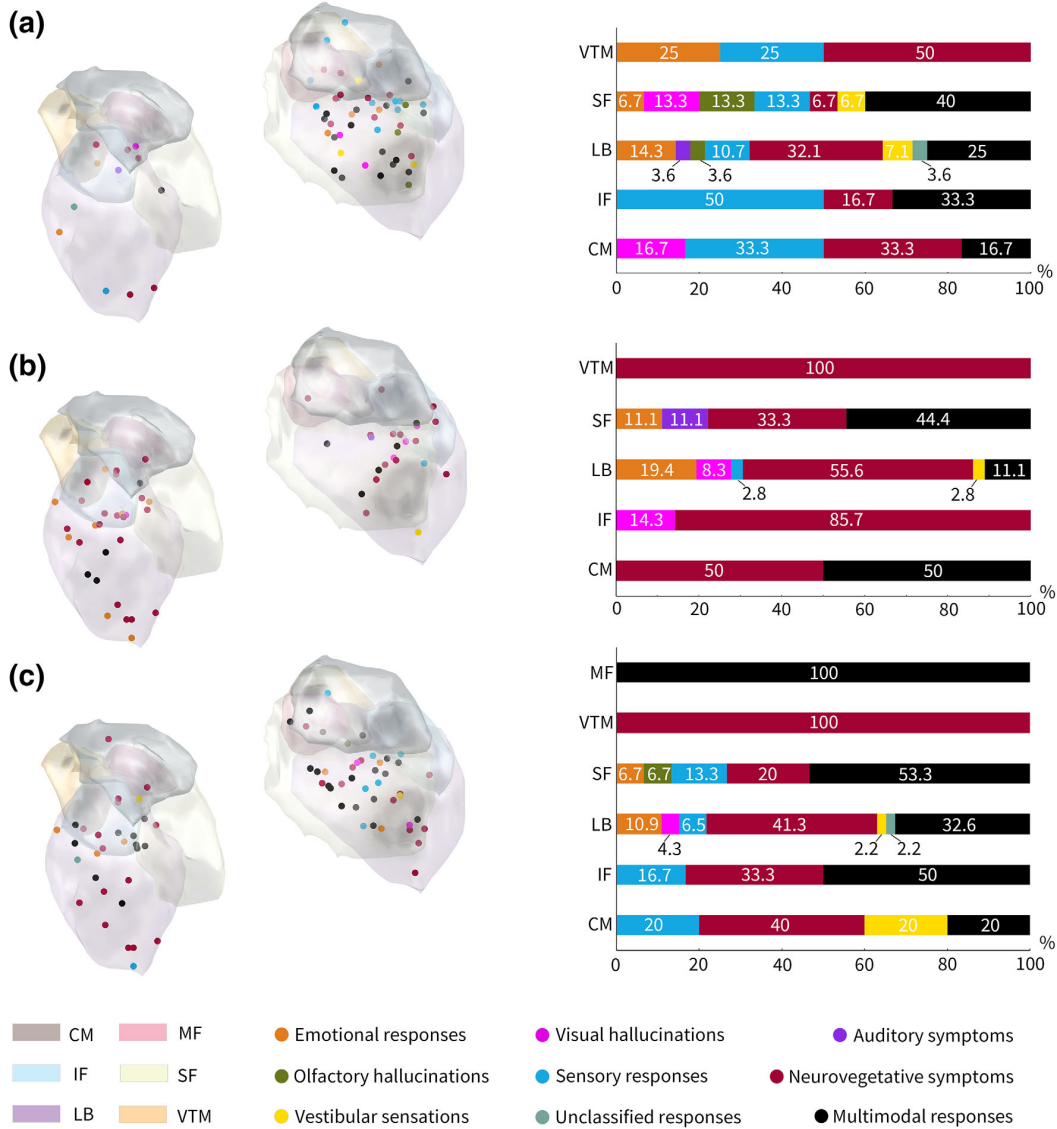
Spatial distribution of contacts within the amygdala (left) and the relative frequencies distribution (right) of elicited responses. In the left column, dots represent stimulation sites in the amygdala. The color of the dots was coordinated with the evoked response distributions in the right column. (a) The distribution of evoked physiological responses without electrical post‐discharge. (b) The distribution of evoked responses related to the usual symptoms but without pathological electric activity. (c) The distribution of evoked responses with electrical after discharge.

Figure 2 depicts the anatomical distribution of all contacts eliciting physiological symptoms and the summary of physiological symptoms obtained in each substructure. Multimodal responses were obtained for 16 contacts (27%, 16/59). Seven (44%, 7/16) of these were in the LB, six (38%, 6/16) in the SF, one (6%, 1/16) in the CM, and two (13%, 2/16) were in the IF. To explore functional anatomy using intracerebral stimulation, multimodal responses were subdivided into each category of physiological symptoms to reduce the possibility of error in interpreting the evoked clinical responses.

#### 3.4.1 Emotional responses

**FIGURE 3:**
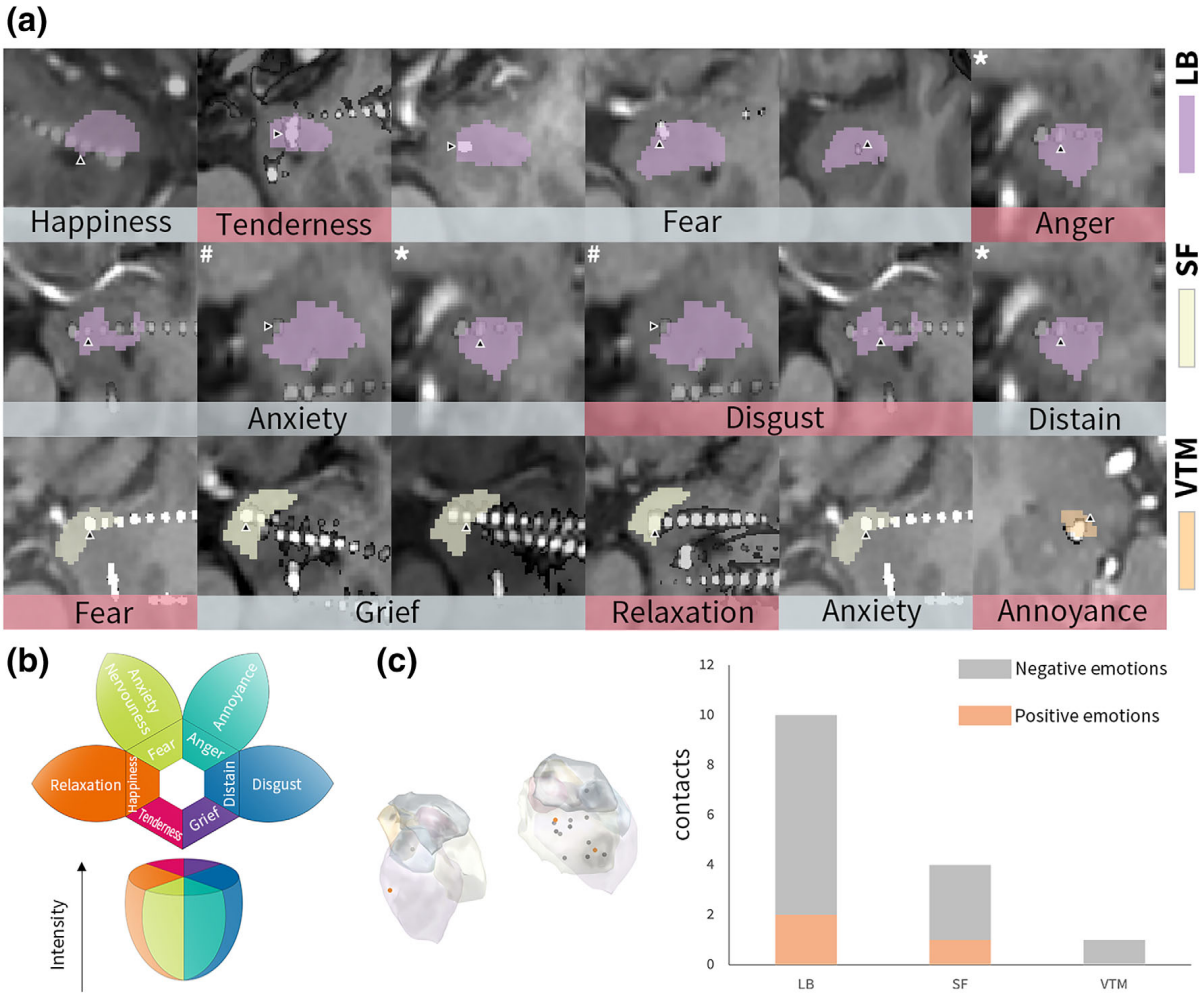
Precise localization of stimulation sites and the evoked emotional responses. (a) The reconstruction of electrodes with postoperative CT images registered in preoperative MRI images. The black triangles indicate the stimulation site. The masks with different colors show the amygdala subnuclei. (b) The emotion wheel of the evoked emotional experiences. (c) The three‐dimensional distribution of stimulation sites of the amygdala with either positive or negative emotions (left) and the emotion distribution in the level of subnuclei (right).

Emotional responses were elicited 25 times in eight patients. Amygdala contacts eliciting emotional responses were asymmetrically distributed across the right and left cerebral hemispheres (3 vs. 12, *χ* ^2^, *p* = .02). Ten (67%, 10/15) of these contacts were in the LB, four (27%, 4/15) in the SF, and one (7%, 1/15) in the VTM (Figure 3).

Emotional responses were classified into positive (three trials) and negative (22 trials) emotions. Positive emotions included happiness, tenderness, and relaxation. Negative emotions included fear, anxiety, nervousness, anger, disdain, disgust, grief, and annoyance. Nervousness was evoked simultaneously with disgust or fear in Patient 35, with one contact in the LB and the other in the SF. Notably, right amygdala stimulation through contact in the LB evoked happiness in Patient 7.

#### 3.4.2 Neurovegetative symptoms

**FIGURE 4:**
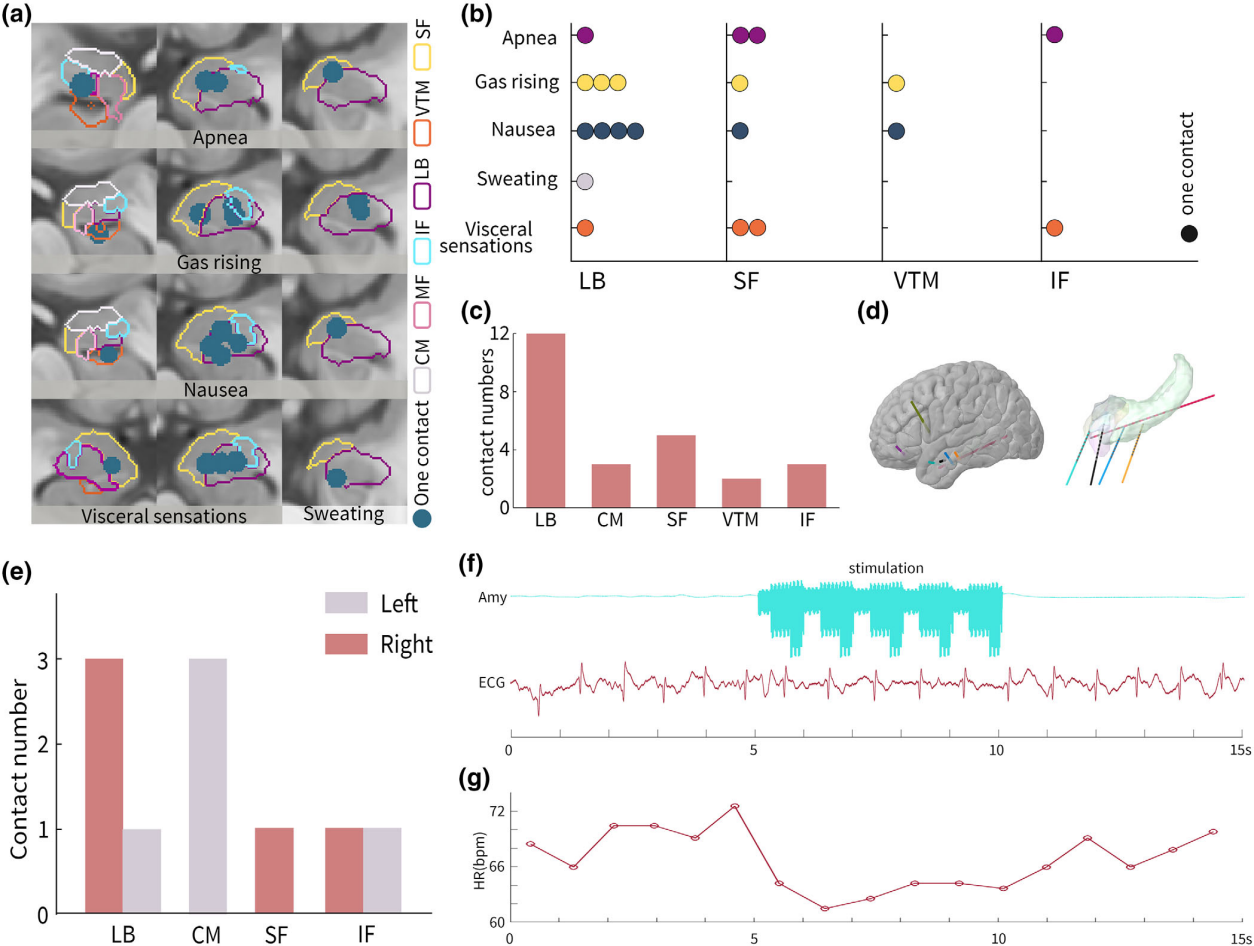
Neurovegetative symptoms evoked by the amygdala subnuclei stimulation. (a) Subnuclei distribution of different neurovegetative symptoms in the MNI space. (b) Subnuclei distribution of evoked symptoms related to the autonomic nervous system. (c) Distribution of stimulation sites within the amygdala subnuclei. (d) The reconstruction of stereoelectroencephalography (SEEG) electrodes with the hemisphere (left) and electrode implanted in the amygdala and hippocampus. (e) Contact numbers of amygdala subnuclei inducing bradycardia in each hemisphere. (f,g) The amygdala stimulation decreased heart rate, displayed with raw data (f) and the temporal heartbeats (g).

Neurovegetative symptoms were elicited 31 times in 14 patients. Amygdala contacts eliciting neurovegetative symptoms were asymmetrically distributed across the right and left cerebral hemispheres (6 vs. 19, *χ* ^2^ *p* = .009). Among these contacts, 12 (48%, 12/25) were in the LB, 5 (20%, 5/25) in the SF, 3 (12%, 3/25) in the CM, 3 (12%, 3/25) in the IF, and 2 (8%, 2/25) in the VTM (Figure 4; Supplementary Figure 2).

Neurovegetative symptoms encompassed several autonomic symptoms, including chest discomfort (10/31, 32%), apnea (5/31, 16%), nausea (8/31, 26%), rising gas (5/31, 16%), sweating (2/31, 6%), and visceral sensations (6/31, 19%). The contact number of amygdala subnuclei inducing bradycardia symptoms was equally five in each hemisphere (Figure 4e). The visceral sensations included burning sensations in the head, instant feeling of thirst, and numbness in part of the pharynx. Different types of neurovegetative symptoms were elicited in five patients simultaneously, with two contacts in the LB, two in the SF, and one in the IF. All CM stimulations induced heart palpitations only.

#### 3.4.3 Visual hallucinations

**FIGURE 5:**
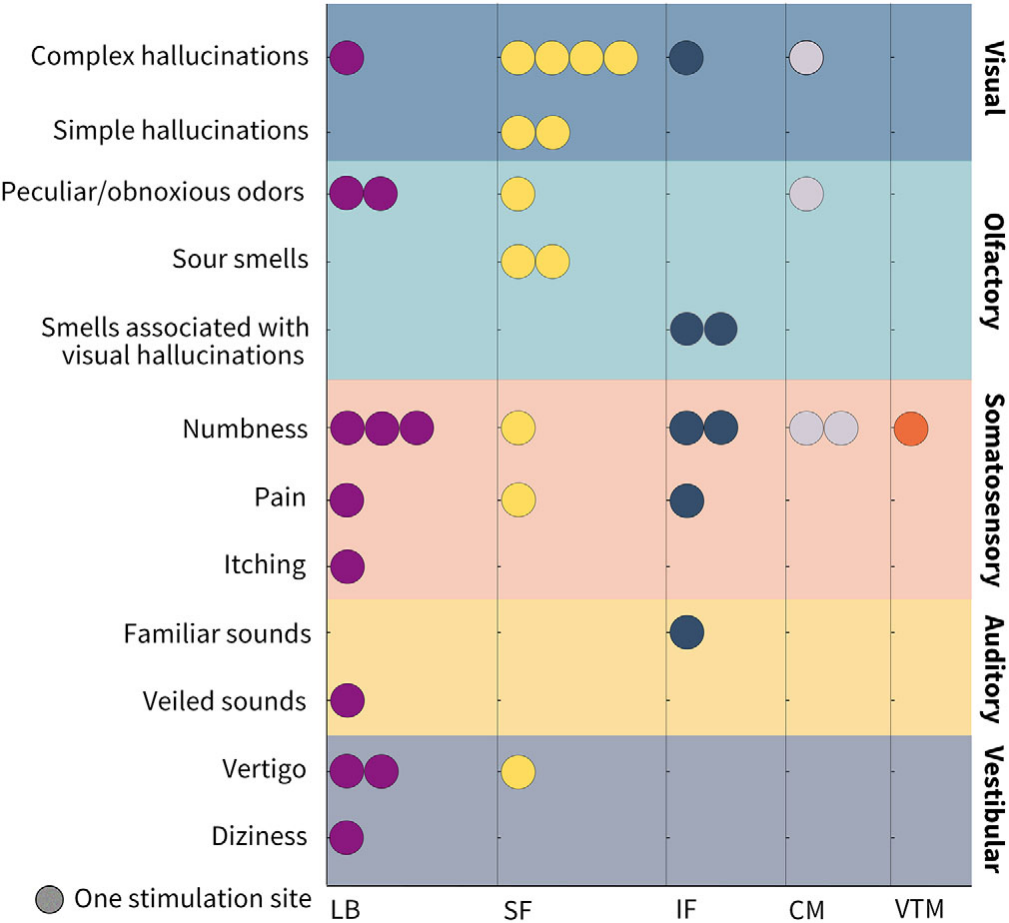
Visual, olfactory, somatosensory, auditory, and vestibular symptoms evoked by the amygdala subnuclei stimulation. The colors of each row correspond to categories of the symptoms on the vertical axis. The circles with colors indicate stimulation sites within the amygdala subnuclei, which are demonstrated on the horizontal axis.

In three patients, visual hallucinations were elicited 18 times. Amygdala contacts eliciting visual hallucinations were found distributed across the right and left cerebral hemispheres (2 vs. 7). One (11%, 1/9) of these contacts was in the LB, six (67%, 6/9) in the SF, one (11%, 1/9) in the CM, and one (11%, 1/9) in the IF (Figure 5). Visual hallucinations include complex and simple forms of visual hallucinations, such as flash dots. Three trials of simple visual hallucinations were elicited by two contacts in the SF of patient 15.

#### 3.4.4 Olfactory hallucinations

Olfactory hallucinations were elicited 12 times in four patients. The amygdala contacts eliciting olfactory hallucinations were distributed in the left cerebral hemisphere (eight contacts). Two (25%, 2/8) of these contacts were in the LB, three (38%, 3/8) in the SF, one (13%, 1/8) in the CM, and two (25%, 2/8) in the IF (Figure 5). Olfactory hallucinations include peculiar/obnoxious odors, sour smells, and characteristic smells associated with visual hallucinations. Peculiar/obnoxious odors include stink, burning, or automobile exhaust. Two sour smell trials were elicited by two contacts in the SF of patient 41. Characteristic smells associated with blood (SF contact) or planks (IF contact) were elicited in patient 32.

#### 3.4.5 Somatosensory responses

Somatosensory responses were elicited 22 times in seven patients. The amygdala contacts eliciting somatosensory responses were found asymmetrically distributed across the right and left cerebral hemispheres (1 vs. 12, *χ* ^2^, *p* = .002). Five (38%, 5/13) of these contacts were in the LB, two (15%, 2/13) in the SF, two (15%, 2/13) in the CM, three (23%, 3/13) in the IF, and one (8%, 1/13) in the VTM (Figure 5). The somatosensory responses included numbness, pain, and itching. Meningeal pain and scalp paresthesia were excluded from the somatosensory responses caused by the propagation of the stimulus to the extracerebral areas. Eleven trials of numbness along the arm or face were elicited in five patients. In patient 33, nine pain trials were elicited in the tooth or the middle of the forehead. The two itching trials were elicited during the SF contact stimulation of patient 35.

#### 3.4.6 Other symptoms

In two patients, auditory symptoms were elicited twice. The two‐amygdala contacts that elicited auditory symptoms were distributed across the right and left cerebral hemispheres. The right contact eliciting veiled sounds was in the LB of patient 37, and the left contact eliciting familiar sounds was in the IF of patient 6.

In three patients, vestibular sensations were elicited five times. The amygdala contacts eliciting vestibular sensations were distributed in the left cerebral hemisphere (four contacts). Three (75%, 3/4) of these were in the LB, and one (25%, 1/4) was in the SF. Vestibular sensations included dizziness elicited by one trial in the LB and vertigo elicited by four trials in the LB or SF.

Unclassified responses, such as vague symptoms, were elicited five times in patients 10 and 32. The amygdala contacts eliciting unclassified responses were distributed across the right and left cerebral hemispheres (one vs. two). Two contacts were in the bilateral LB, and one was in the left SF.

### 3.5 Subnucleus distribution of clinical symptoms

**FIGURE 6:**
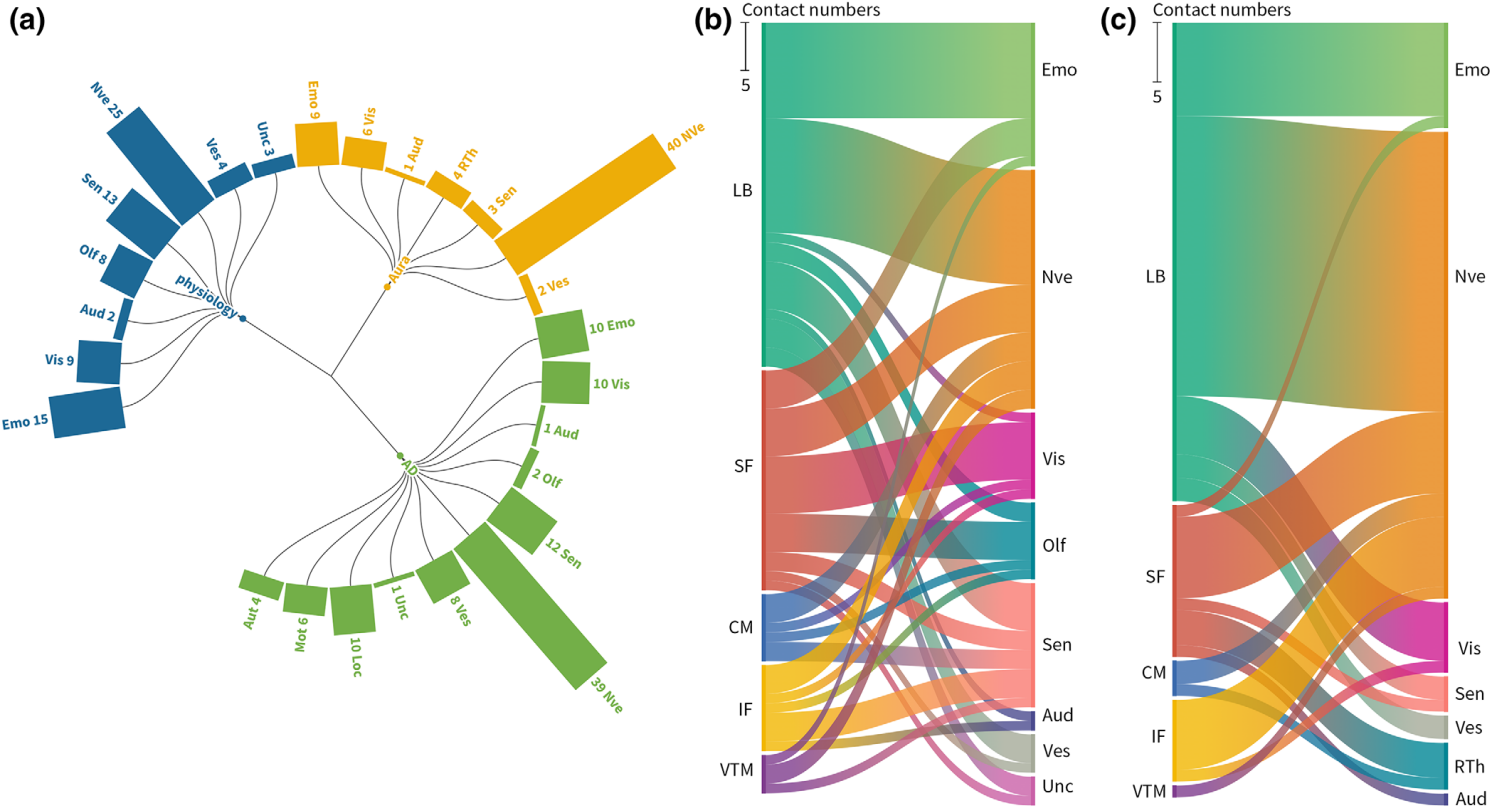
Categories of responses and the distribution of amygdala stimulation sites with different responses. (a) Contact numbers of each category in physiological responses, usual symptoms without pathological electrical activity, and electrical after discharge. (b) The distribution of stimulation sites with different physiological responses across the amygdala subnuclei. (c) The distribution of stimulation sites with responses related to habitual aura symptoms.

Physiological symptoms were divided into eight categories (Figure 6). The percentage distribution of each physiological symptom category for each subnucleus revealed that the most frequently evoked neurovegetative symptoms were distributed in almost all subnuclei. LB is mainly associated with emotional responses, somatosensory responses, and vestibular sensations. SF is mainly associated with emotional responses, visual hallucinations, and olfactory hallucinations. Both CM and IF are associated with somatosensory responses.

In 21 patients, usual symptoms without pathological electrical activity were observed. The localization of these contact electrodes revealed 36 contacts (65%, 36/55) in the LB, 9 (16%, 9/55) in the SF, 2 (4%, 2/55) in the CM, 7 (13%, 7/55) in the IF, and 1 (2%, 1/55) in the VTM. All CM stimulations induced only heart palpitations. Among the usual symptoms without pathological electric activity, multimodal responses were obtained in nine contacts (16%, 9/55). The percentage distribution of each usual symptom category for each subnucleus revealed that the most frequently evoked neurovegetative symptoms were distributed in most subnuclei. LB was mainly associated with emotional responses and visual hallucinations. SF was mainly associated with racing thoughts (Figure 6).

Clinical symptoms associated with AD were observed in 31 patients. The localization of these contact electrodes revealed 46 contacts (62%, 46/74) in the LB, 15 (20%, 15/74) in the SF, 5 (7%, 5/74) in the CM, 6 (8%, 6/74) in the IF, 1 (1%, 1/74) in the VTM, and 1 (1%, 1/74) in the MF. Multimodal responses were observed in 28 patients (38%, 28/74) with clinical symptoms associated with AD. The percentage distribution of each category of clinical symptoms associated with AD for each subnucleus revealed that, except for MF, the most frequently evoked neurovegetative symptoms were distributed in every subnucleus. In addition, LB is mainly associated with emotional and somatosensory responses. SF was mainly associated with vestibular sensations (Supplementary Figure 1). In two patients, stimulation of the SF and IF caused uncinate seizures. One of these two patients experienced obnoxious odors when the ictal activity occurred after SF stimulation. The other patient felt the smell of the earth. Then, he reported seeing something in the familiar shape of a cylinder following epileptic discharges caused by the IF simulation.

Overall, 81 contacts in the amygdala subnuclei elicited seizures. Fifty‐four (67%, 54/81) of these were in the LB, thirteen (16%, 13/81) in the SF, four (5%, 4/81) were in the CM, seven (9%, 7/81) in the IF, two (2%, 2/81) in the VTM, and one (1%, 1/81) in the MF.

Statistical stronger stimulation intensity was found in the after discharge (AD) and usual symptoms without AD, compared with the intensity of the physiological symptom. The stimulation current intensity of eight main categories based on the characterized profiles of the elicited physiological responses was 1.93 ± 0.92 mA (emotional responses), 1.95 ± 1.27 mA (neurovegetative symptoms), 2.06 ± 1.54 mA (visual hallucinations), 3.1 ± 2.34 mA (olfactory hallucinations), 1.79 ± 1.24 mA (somatosensory responses), 3.5 ± 0.71 mA (auditory symptoms), 1.88 ± 1.21 mA (vestibular sensations), and 2.24 ± 1.76 mA (unclassified responses), respectively (Supplementary Figure 3).

### 3.6 Amygdala‐cortical connectivity map determined by intracranial electrical evoked responses

Amygdala‐cortical evoked potentials (ACEPs) were used to evaluate the direct electrophysiological connectivity between the amygdala and the cortex in vivo. In 10 patients, contact implantation into the amygdala was used to deliver repetitive single‐pulse direct electrical stimulation. Further, while the morphology of the cortical evoked responses may vary, an early sharper biphasic potential component (classically termed N1, peak around 10–50 ms), and a later slow‐wave‐like potential component (classically termed N2, peak around 50–200 ms) has been used to describe evoked neural activity qualitatively reliably. In addition, different cortical areas respond differently to amygdala stimulation.

**FIGURE 7:**
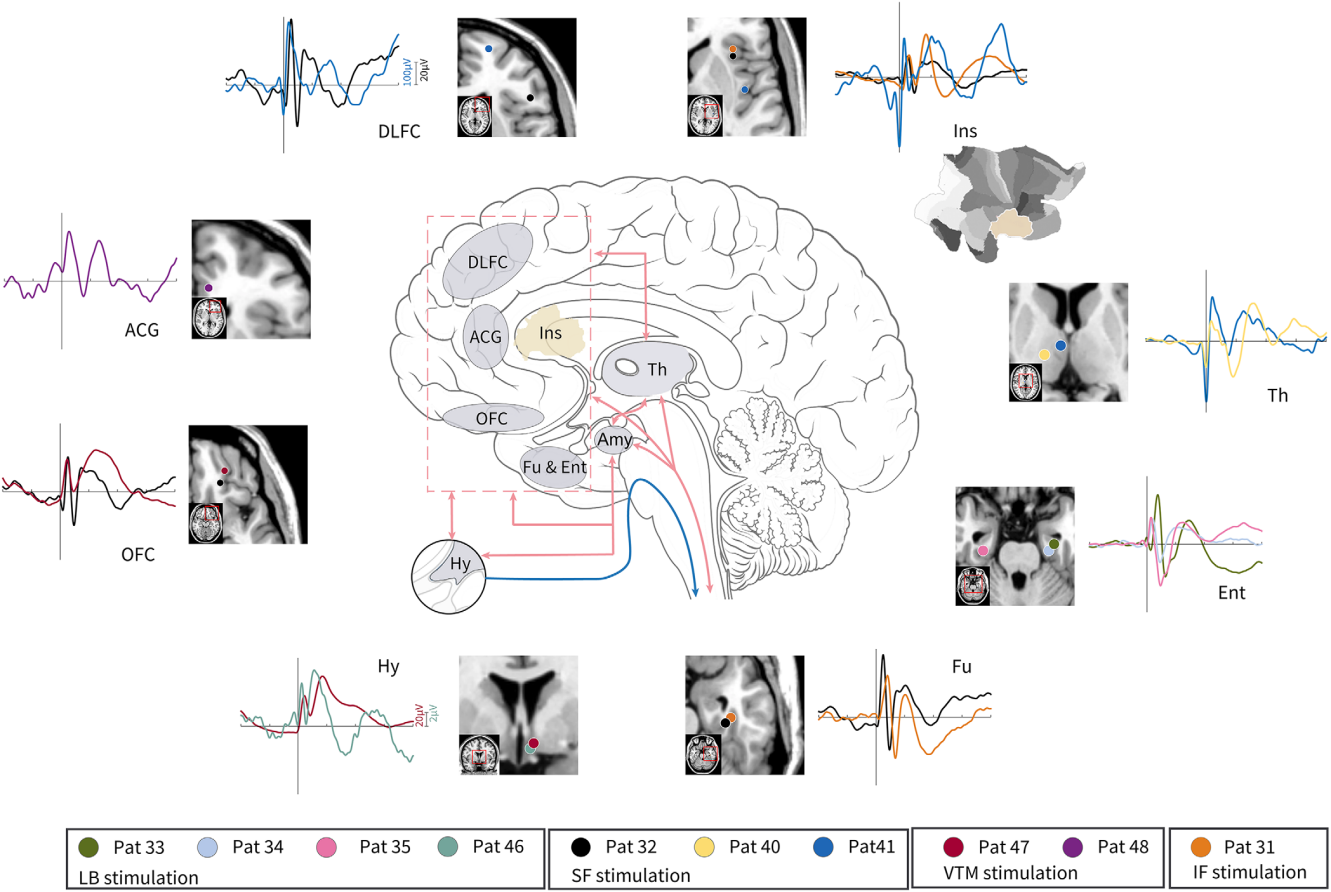
Overall circuits revealed by amygdala‐cortical evoked potential responses in 10 patients who have received amygdala‐cortical evoked investigation. Each dot over the slices represents a site with significant response potential evoked by amygdala stimulation. A different color identifies the evoked response of each patient consistent with the color shown in the dot. The red and blue arrows indicate information flows.

There were 17 contacts in the gray matter without significant artifacts or epileptic discharges. Fifteen of these were ipsilateral to the side of amygdala implantation, and two were contralateral, one in the hypothalamus (patient 46) and another in the cingulate gyrus (patient 48). The root mean square (RMS) values of 50 non‐averaged post‐stimulation epochs in the early (10–50 ms) and later (50–200 ms) time windows were separately compared with the RMS value of 50 non‐averaged pre‐stimulation epochs (−200 to −50 ms) using the paired two‐sample *t* test to measure the magnitude of evoked neural responses for each cortical contact site. In comparison, the spatially distributed pattern of ACEP‐based connectivity revealed that the amygdala is closely connected to the hypothalamus, anterior nucleus of the thalamus, cingulate gyrus, entorhinal cortex, fusiform gyrus, orbitofrontal cortex, dorsolateral frontal cortex, and insula in both early and later time windows (Figure 7).

## 4 DISCUSSION

This study provides a useful picture of direct electrical stimulation of human subnuclei. This direct “casual‐effect” perturbation approach was used in a relatively large patient group to provide a reliable, functional map. Compared to most intracranial stimulation studies on the role of the amygdala in emotional experience, the main findings of this study revealed that the human amygdala plays a vital role beyond the exclusive processing of emotional experience. Further, it participates in the autonomic regulation and sensory functions of the body at the subnuclei level: (a) the amygdala was found not to mediate basic emotions specifically. However, it was involved in more general and intrinsic brain networks mediating multiple emotions. (b) Evidence revealed that the CM and other subnuclei were implicated in autonomic nervous system activity. (c) The amygdala is suggested to be involved in multimodal sensory processing, including somatosensory, visual, auditory, and vestibular sensory processing.

A key novel finding of this study was that direct amygdala stimulation could elicit several emotions related to unpleasantness and pleasure in both the left and right amygdala subnuclei. To date, a point of debate in the neural representation of emotion is whether the amygdala specifically mediates basic emotions or plays a crucial role in multiple emotions (Gu et al., 2019; Hamann, 2012). A strong relationship between the human amygdala and basic emotions has been revealed in studies of intracranial amygdala stimulation. These studies revealed that amygdala stimulation elicited responses of fear, anxiety, sadness, and joy, with the right hemisphere stimulation producing negative responses but the left hemisphere producing both positive and negative emotions (Bijanki et al., 2014; Inman et al., 2020; Lanteaume et al., 2007; Meletti et al., 2006). Negative emotional responses were induced more frequently than positive emotional experiences, which is consistent with previous research. However, the emotions of disdain, grief, and tenderness induced by direct amygdala stimulation in this study have not been previously described. In this study, negative and positive emotions were evoked in both hemispheres. This is consistent with several fMRI meta‐analyses that showed no amygdala lateralization based on stimuli valence (Baas et al., 2004). BLA stimulation has been revealed to trigger fear‐related responses in the amygdala subnuclei (Inman et al., 2020). In this study, the BLA was also related to happiness, tenderness, disgust, and disdain. SF stimulation is associated with fear, grief, relaxation, and anxiety. It can be assumed that the amygdala is involved in general and intrinsic brain networks mediating multiple emotions, regardless of lateralization to the left or right hemispheres.

The amygdala, anterior cingulate cortex, insular cortex, thalamus, hypothalamus, periaqueductal grey matter, parabrachial nucleus, and several medullary regions—regulate the central processing of sympathetic and parasympathetic outflow (Thijs et al., 2021). However, few studies have investigated the effects of intracranial stimulation on the ANS. Previous studies have revealed that amygdala stimulation induces apnea (Nobis et al., 2018) and bradycardia (Inman et al., 2020). Inman CS et al. found that the incidence of bradycardia was equal between the left and right amygdala, but left amygdala stimulation would be predicted to elicit −2.18 beats per minute greater deceleration in heart rate than the right amygdala (Inman et al., 2020). Chouchou et al. reported that the amygdala and the simulation resulted in heart rate increments in heroin addicts (Chouchou et al., 2019). Sanchez‐Larsen found no significant correlation between amygdala stimulation and cardiovascular functions (Sanchez‐Larsen et al., 2021). These different results may occur due to the bias of the limited stimulation sites. Another explanation is that the amygdala subnuclei have mixed effects on the heart rate. Nonetheless, because these studies did not focus on this issue, amygdala subdivision that induces changes in heart rate remains unclear. This study also revealed that LB, SF, and IF stimulation elicited apnea and bradycardia. Additionally, CM and VTM stimulation induced bradycardia. Previous studies on amygdala stimulation did not report symptoms of rising gas, nausea, visceral sensations, or sweating. Thus, the human amygdala plays a vital role in the ANS, controlling respiratory, cardiovascular, and gastrointestinal functions.

In this study, one important finding was that amygdala stimulation induced olfactory, somatosensory, auditory, visual, and vestibular sensations. The SF in rodents receives inputs from the main olfactory bulb, however, not the accessory olfactory bulb, suggesting its important role in the main olfactory system (McDonald, 1998). The olfactory bulb projects directly to the SF (also called the periamygdaloid complex) in humans(Allison, 1954; Crosby & Humphrey, 1941). SF stimulation produces olfactory seizures, indicating SF involvement in the olfactory function system. In addition, whole‐brain functional network analysis revealed strong connectivity between the SF and the temporal pole, fusiform cortex, hippocampus, parahippocampal gyrus, orbitofrontal cortex, entorhinal cortex, and dorsal pons (Noto et al., 2021). These findings suggest that projections from the olfactory and uncinate fasciculus exist in the SF. Little is known about IF role in olfactory information processing because of limited studies on it. IF stimulation produced smells associated with visual hallucinations, which could be caused by seizure spread. According to findings from amygdala stimulation in patients with schizophrenia and epilepsy reported by Ishibashi et al. (1964) and Mariani et al. (2021), amygdala stimulation produced simple visual hallucinations in these studies. Studies have revealed the involvement of the amygdala in visual information processing; however, whether the amygdala directly participates in the visual pathway remains unknown. Therefore, it should be investigated further.

Previous studies have revealed that the amygdala receives a wide range of inputs from cortical and subcortical regions, which are involved in information processing and emotion generating (Diano et al., 2016). The BLA is generally regarded as the gatekeeper for sensory inputs from the visual, auditory, somatosensory, olfactory, and vestibular systems (LeDoux, 2007). In addition, other amygdala regions receive inputs from the BLA and other brain areas, allowing the amygdala to process a wide range of information. Intracranial recordings of the human amygdala response to sensory stimuli have revealed a similar timing of auditory and visual sensory information (Dominguez‐Borras et al., 2019). However, both afferent inputs into the amygdala and neuronal membrane potential may be reflected in the evoked response potential during intracranial recordings. Another important finding in this study was that amygdala stimulation induced visual, auditory, somatosensory, olfactory, and vestibular sensations. The ACEP in our study has also revealed that the amygdala is functionally connected with the prefrontal cortex, anterior cingulate cortex, hypothalamus, fusiform cortex, entorhinal cortex, insular cortex, and thalamus, which is consistent with findings of previous studies. This highlights the direct involvement of the amygdala in sensory processing, autonomic regulation, and emotion generation in humans.

This study had some limitations. First, all enrolled patients had drug‐resistant epilepsy; thus, their brain structures and functions are atypical, necessitating cautiousness in relating and interpreting the findings of this study in patients with healthy brains. Second, evoked clinical manifestations are subjective and dependent on the collaboration and cognitive function of the patients. Third, the lack of quantified monitoring of emotional states and ANS function, such as blood pressure and respiration measurements, limited reliability. Finally, the topographical mapping of the functional architecture of the amygdala was constrained by incomplete and uneven coverage because only a limited number of electrodes can be placed within the amygdala subnuclei based on clinical needs. However, the study provides a valuable profile of the amygdala subnuclei in humans, which should be a reference point in future research and clinical intervention in patients with psychiatric illnesses involving the amygdala.

## 5 CONCLUSION

The novelty of this study with direct electrical stimulation of the human amygdala subnuclei reveals that the human amygdala is not only involved in basic emotions but also in more general multiple emotions. Further, the human amygdala plays an essential role in the ANS in controlling respiratory, cardiovascular, and gastrointestinal functions at the subnuclei level. The sensory symptoms elicited by amygdala stimulation highlight the direct link between the amygdala and multiple sensory processes in humans. Our findings led to the development of a functional map of the human amygdala for emotion generation, ANS regulation, and sensory information processing.

## CONFLICT OF INTEREST

The authors declare no conflict of interest.

## Supporting information


**SUPPLEMENTARY FIGURE 1**. Categories of responses and the distribution of amygdala stimulation sites linked with electrical after discharge. Abbreviations: LB, laterobasal group; SF, superficial group; CM, centromedial group; IF, intermediate fiber bundles; VTM, ventromedial part; MF, medial fiber bundles; Emo, emotional responses; Nve, neurovegetative responses; Vis, visual responses; Olf, olfactory responses; Sen, somatosensory responses; Aud, auditory responses; Ves, vestibular responses; Loc, loss of contact; Mot, motor symptoms; Aut, automatisms; Unc, unclassified responses.

**SUPPLEMENTARY FIGURE 2**. Amygdala stimulation sites of neurovegetative symptoms. The teal green masks indicate the location of stimulation sites. The outlines with different colors indicate the amygdala subnuclei.

**SUPPLEMENTARY FIGURE 3**. Stimulation intensity. (A) There was a statistical significance of stimulation intensity between physiological responses and usual responses or after discharges. (B) Violin plot of stimulation intensity of different response types in physiological responses. Abbreviations: Emo: emotion, Nve: neurovegetative, Vis: visual, Olf: olfactory, Sen: somatosensory, Aud: auditory, Ves: vestibular, Unc: unclassified.

**SUPPLEMENTARY TABLE 1**. Detailed clinical profiles of each patient.

**SUPPLEMENTARY TABLE 2**. Main definitions used for the classification of the responses in the study.

## Data Availability

Data sharing is not applicable to this article as no new data were created or analyzed in this study.
