# Supplementary figures and images for: Integrative roles of human amygdala subdivisions: Insight from direct intracerebral stimulations via stereotactic EEG
Source: Hum Brain Mapp. 2023 Apr 19;44(9):3610–23. doi: 10.1002/hbm.26300 (PMC10203795; doi:10.1002/hbm.26300)

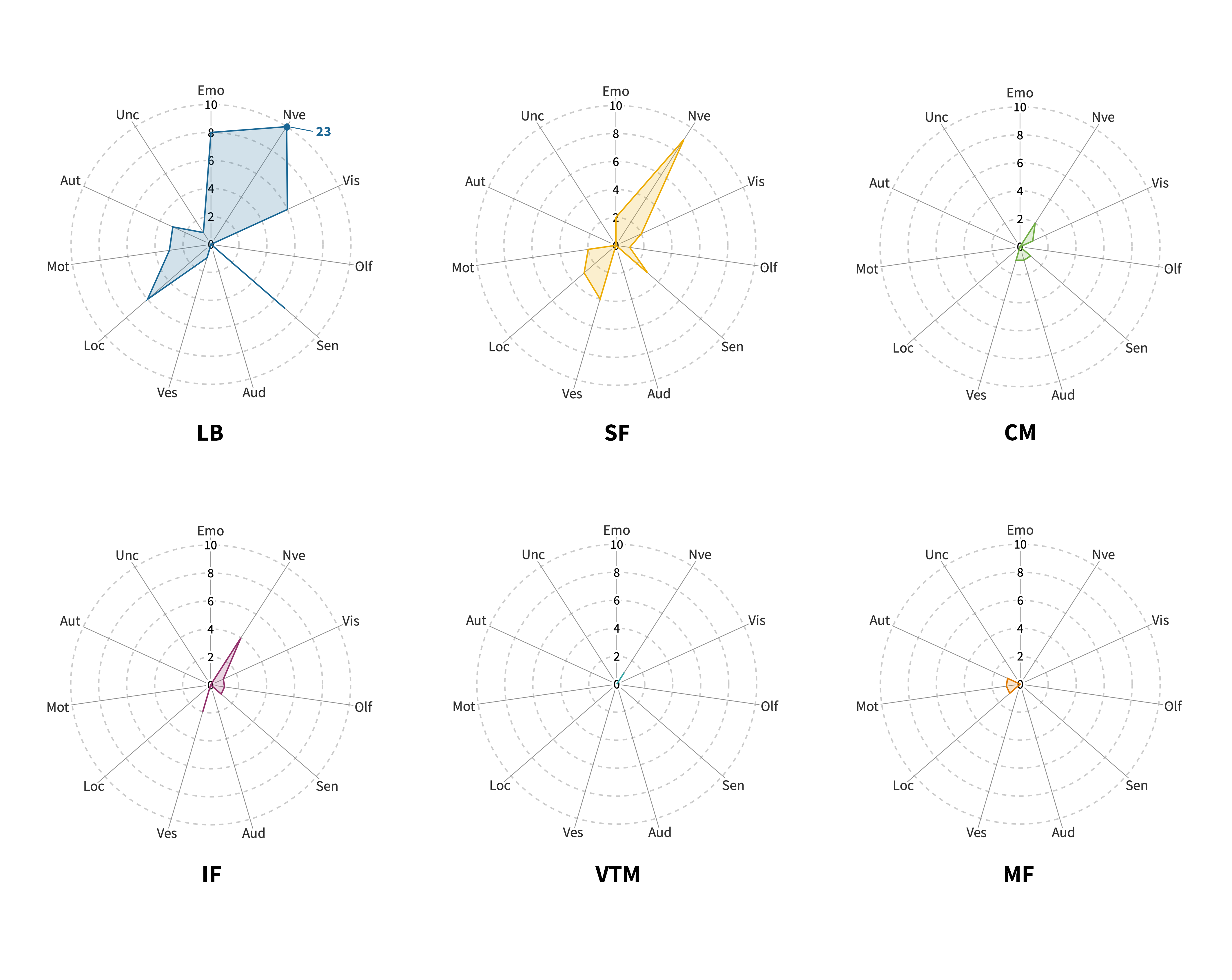

Supplement: Supplementary file 1 — SUPPLEMENTARY FIGURE 1. Categories of responses and the distribution of amygdala stimulation sites linked with electrical after discharge. Abbreviations: LB, laterobasal group; SF, superficial group; CM, centromedial group; IF, intermediate fiber bundles; VTM, ventromedial part; MF, medial fiber bundles; Emo, emotional responses; Nve, neurovegetative responses; Vis, visual responses; Olf, olfactory responses; Sen, somatosensory responses; Aud, auditory responses; Ves, vestibular responses; Loc, loss of contact; Mot, motor symptoms; Aut, automatisms; Unc, unclassified responses. [file HBM-44-3610-s002.png]

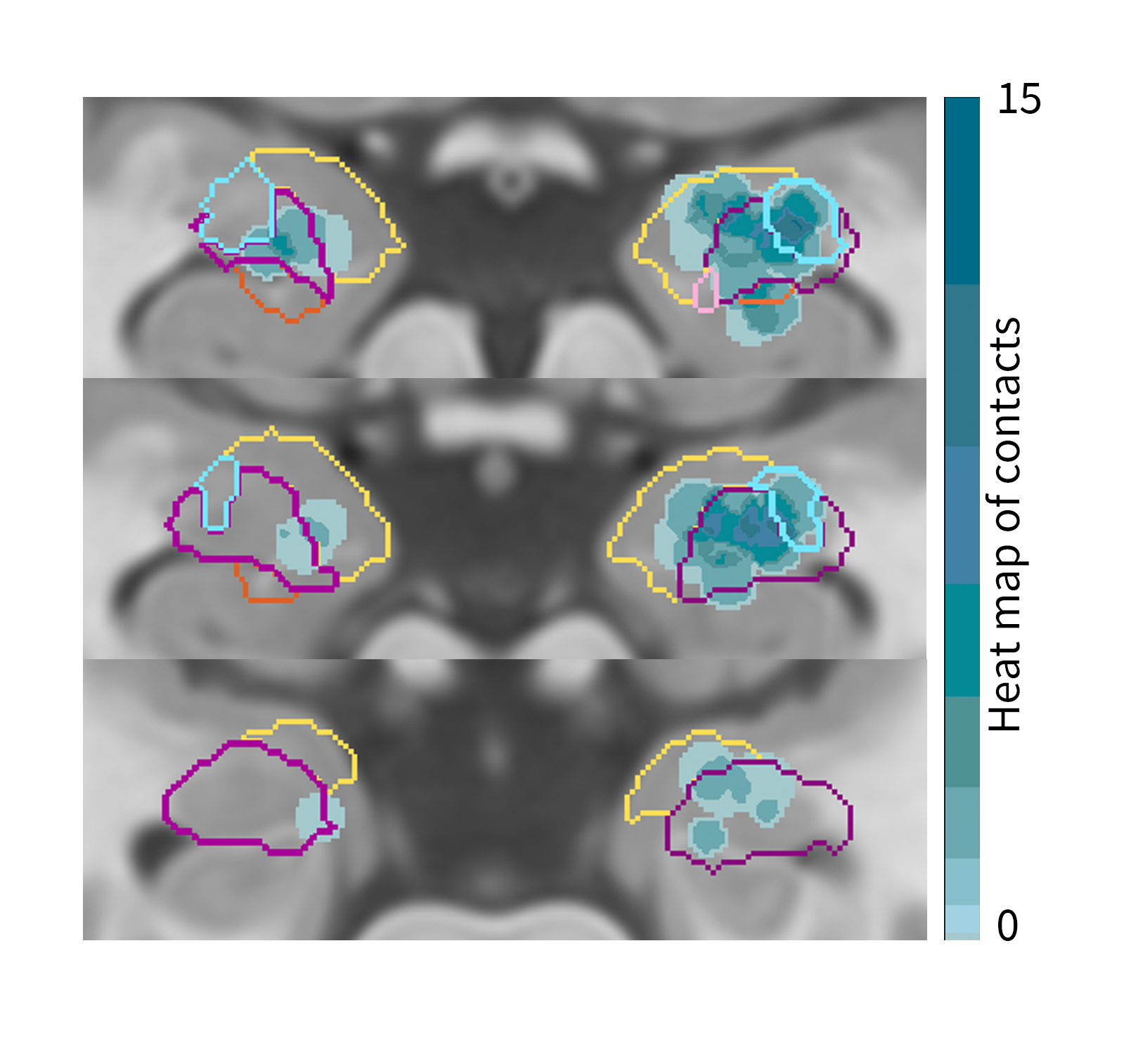

Supplement: Supplementary file 2 — SUPPLEMENTARY FIGURE 2. Amygdala stimulation sites of neurovegetative symptoms. The teal green masks indicate the location of stimulation sites. The outlines with different colors indicate the amygdala subnuclei. [file HBM-44-3610-s003.png]

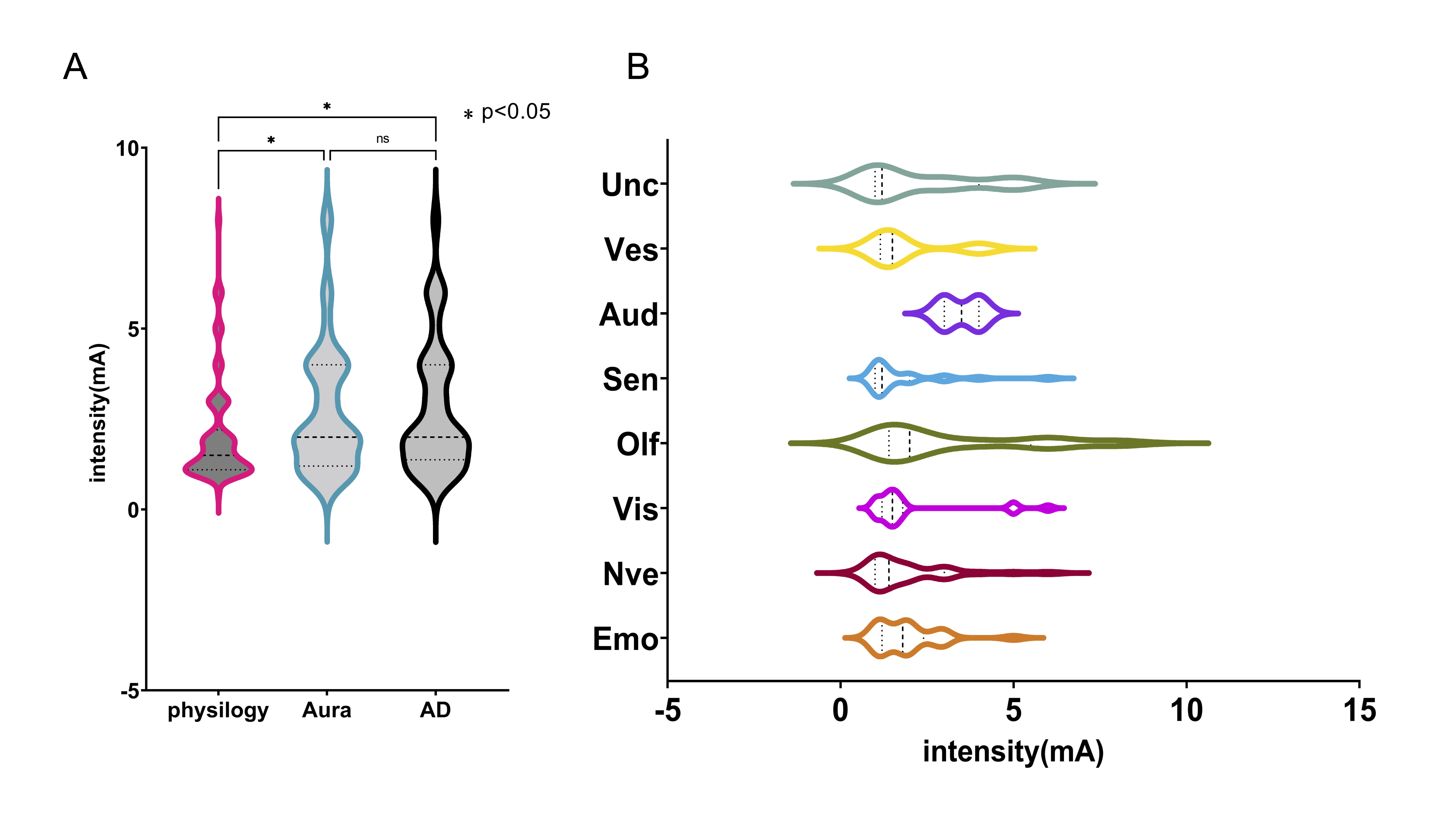

Supplement: Supplementary file 3 — SUPPLEMENTARY FIGURE 3. Stimulation intensity. (A) There was a statistical significance of stimulation intensity between physiological responses and usual responses or after discharges. (B) Violin plot of stimulation intensity of different response types in physiological responses. Abbreviations: Emo: emotion, Nve: neurovegetative, Vis: visual, Olf: olfactory, Sen: somatosensory, Aud: auditory, Ves: vestibular, Unc: unclassified. [file HBM-44-3610-s004.png]
