# Supplementary material for: Integrative roles of human amygdala subdivisions: Insight from direct intracerebral stimulations via stereotactic EEG
Source: Hum Brain Mapp. 2023 Apr 19;44(9):3610–23. doi: 10.1002/hbm.26300 (PMC10203795; doi:10.1002/hbm.26300)
Supplement: Supplementary file 4 — SUPPLEMENTARY TABLE 1. Detailed clinical profiles of each patient. [file HBM-44-3610-s005.docx]

**Supplementary table 1**

| Patient | Sex | Handness | Age  (y) | Duration  (y) | Aura | SOZ | Side of SEEG | | Contacts within amygdala  Total(L/R) | Number of trials  Total(L/R) |
| --- | --- | --- | --- | --- | --- | --- | --- | --- | --- | --- |
|  | M | R | 26 | 19 | headache | R-mTL | R | 1 | | 1 |
|  | M | R | 19 | 4 | - | L-mTL | L | 1 | | 1 |
|  | M | R | 19 | 10 | auditory hallucinations | L-INS | L | 1 | | 1 |
|  | F | R | 21 | 6 | fear, nervousness, hot flushes | R-mTL | R | 2 | | 4 |
|  | M | R | 21 | 20 | nausea | L-mTL | L | 5 | | 5 |
|  | F | R | 16 | 11 | tinnitus | L-INS | L | 2 | | 2 |
|  | M | R | 49 | 15 | epigastric upset, epigastric rising, déjà vu | R-mTL | R | 1 | | 1 |
|  | M | R | 32 | 15 | - | R-mTL | R | 2 | | 3 |
|  | F | R | 17 | 8 | epigastric rising | L-mTL | L | 3 | | 3 |
|  | M | R | 37 | 11 | chest discomfort | R-mTL | Bi | 5(1/4) | | 7(1/6) |
|  | F | R | 29 | 19 | - | L-mTL | L | 6 | | 6 |
|  | F | R | 27 | 11 | Chest tightnes | L-mTL | L | 3 | | 4 |
|  | M | R | 53 | 10 | heart palpitation | BTL | Bi | 6(4/2) | | 8(5/3) |
|  | M | R | 57 | 40 | apnea | L-mTL | Bi | 3(2/1) | | 3(2/1) |
|  | F | R | 42 | 2 | - | R-TLp | Bi | 3(1/2) | | 5(1/4) |
|  | F | R | 28 | 17 | epigastric upset, heart palpitation | R-mTL | R | 3 | | 3 |
|  | M | R | 15 | 1 | headache, chest tightness | L-FL | L | 2 | | 2 |
|  | M | R | 30 | 15 | body numbness,epigastric rising | R-mTL | R | 2 | | 7 |
|  | F | R | 25 | 12 | epigastric upset, heart palpitation | R-mTL | R | 3 | | 3 |
|  | F | R | 27 | 13 | Chest tightnes | R-mTL | L | 1 | | 1 |
|  | M | R | 36 | 8 | heart palpitation | R-mTL | R | 6 | | 13 |
|  | M | R | 27 | 18 | fear | R-mTL | R | 6 | | 11 |
|  | F | R | 23 | 16 | déjà vu, salivation,heart palpitation, nausea, epigastric rising | L-mTL | L | 1 | | 1 |
|  | M | R | 22 | 8 | heart palpitation,nausea, salivation, headache | R-mTL | R | 3 | | 3 |
|  | M | R | 18 | 3 | dizziness, nausea | L-mTL | L | 1 | | 1 |
|  | M | R | 29 | 20 | Visual blurring, heart palpitation | R-mTL | R | 3 | | 3 |
|  | F | R | 13 | 4 | vague auras | R-mTL | R | 1 | | 1 |
|  | F | R | 23 | 10 | - | R-mTL | R | 1 | | 1 |
|  | M | R | 21 | 7 | - | L-FL | L | 2 | | 3 |
|  | M | R | 17 | 10 | fear, heart palpitation | L-mTL | L | 5 | | 7 |
|  | F | R | 35 | 34 | lightheaded, heart palpitation, epigastric rising | L-mTL | L | 6 | | 14 |
|  | M | R | 22 | 13 | epigastric rising, déjà vu | L-mTL | L | 8 | | 31 |
|  | F | R | 41 | 20 | - | L-mTL | L | 4 | | 13 |
|  | F | R | 26 | 11 | nausea | R-mTL | R | 7 | | 15 |
|  | F | R | 39 | 30 | déjà vu | L-mTL | L | 5 | | 22 |
|  | M | R | 27 | 13 | unable to understand spoken words, heart palpitation | L-mTL | L | 5 | | 5 |
|  | F | R | 39 | 9 | - | L-TL | Bi | 4(3/1) | | 6(5/1) |
|  | F | R | 28 | 25 | dreaming, déjà vu | L-mTL | Bi | 4(3/1) | | 4(3/1) |
|  | M | R | 31 | 23 | fear, chest tightness | L-opercula | L | 3 | | 3 |
|  | M | R | 24 | 4 | nausea | L-mTL | Bi | 2(1/1) | | 2(1/1) |
|  | F | R | 19 | 10 | Pharyngeal dysesthetic, epigastric rising | L-TL | L | 2 | | 2 |
|  | M | R | 31 | 29 | Racing thoughts，heart palpitation | L-FL&TL | L | 5 | | 5 |
|  | F | R | 31 | 17 | - | L-FL | L | 1 | | 1 |
|  | M | R | 18 | 11 | numbness of head, anxiety | R-SMA | R | 2 | | 2 |
|  | F | R | 31 | 16 | déjà vu | L-TL | L | 4 | | 5 |
|  | F | R | 28 | 23 | heart palpitation, chest tightness, nervousness, déjà vu | HH | Bi | 4(2/2) | | 4(2/2) |
|  | M | R | 19 | 16 | - | HH | L | 1 | | 1 |
|  | M | R | 43 | 42 | dizziness | L-FL | R | 1 | | 1 |
