# Supplementary material for: Integrative roles of human amygdala subdivisions: Insight from direct intracerebral stimulations via stereotactic EEG
Source: Hum Brain Mapp. 2023 Apr 19;44(9):3610–23. doi: 10.1002/hbm.26300 (PMC10203795; doi:10.1002/hbm.26300)
Supplement: Supplementary file 5 — SUPPLEMENTARY TABLE 2. Main definitions used for the classification of the responses in the study. [file HBM-44-3610-s001.docx]

***Supplementary Table 2***

*Main definitions used for the classification of the responses in the study.*

| *Glossary* |
| --- |
| *Physiological symptoms refer to responses never experienced by the patient before and* *without electrical modification* |
| *Usual symptoms refer to responses resemble the usual ictal symptomatology and is recognized by the patient and/or the habitual witnesses of the seizures* |
| *An after discharge (AD) refers to ‘any rhythmic activity on the EEG that is different from the pre-stimulation activity, starting during or immediately after the stimulation, with no time interval from the end of the stimulation, and lasting at least one second’ (Fish et al., 1993).* |

*Ref.*

*Fish, D. R., Gloor, P., Quesney, F. L., & Oliver, A. (1993). Clinical responses to electrical brain stimulation of the temporal and frontal lobes in patients with epilepsy: Pathophysiological implications. Brain, 116(2), 397–414. https://doi.org/10.1093/ brain/116.2.397*
